# Complete Genome Sequence of *Pseudoxanthomonas suwonensis* Strain J1, a Cellulose-Degrading Bacterium Isolated from Leaf- and Wood-Enriched Soil

**DOI:** 10.1128/genomeA.00614-15

**Published:** 2015-06-11

**Authors:** Liyuan Hou, Jingwei Jiang, Zhihui Xu, Yun Zhou, Frederick Chi-Ching Leung

**Affiliations:** a Bioinformatics Centre, Nanjing Agricultural University, Nanjing, China; b School of Biological Sciences, the University of Hong Kong, Hong Kong SAR, China

## Abstract

We report here the complete genome sequence of the cellulose-degrading bacterium *Pseudoxanthomonas suwonensis* strain J1, isolated from soil enriched with rotten leaves and wood from the Zhong Mountain Scenic Area in Nanjing, China. This complete genome may contribute to further investigation of plant biomass degradation.

## GENOME ANNOUNCEMENT

Cellulose, an abundant and renewable natural resource, has great potential to become a biofuel that can help solve the global energy issue (1). Soil on mountains with plenty of wood and leaves contains many kinds of microorganisms that can degrade fallen leaves and wood. Strain J1 is a Gram-negative and non-spore-forming rod bacterium, which was isolated from soil enriched by rotten leaves and wood on Zhong Mountain, China. The colonies of strain J1 are round, yellow, and convex, with entire margins (2). J1 can grow in mesophilic and aerobic environments, utilizing carboxymethylcellulose (CMC) as a sole carbon source. Through filter paper and carboxymethyl cellulase assays, the strain has been shown to have high cellulase activity (3). The 16S rRNA gene sequence of strain J1 shows 99% identity to *Pseudoxanthomonas suwonensis* strain 11-1. The genome sequencing may be helpful in further research and development of the cellulase of the *Pseudoxanthomonas* genus.

Strain J1 was cultured from pure aerobic medium with CMC as the sole carbon source. Genomic DNA was extracted using a bacterial DNA kit (Omega) and quantified using a NanoDrop 2000 spectrophotometer (Thermo Scientific) and a Quant-iT PicoGreen double-stranded DNA (dsDNA) kit (Invitrogen). Whole-genome sequencing of strain J1 was performed with the Illumina MiSeq platform (Illumina) at the Nanjing Agricultural University Bioinformatics Centre, Jiangsu Province, China. The shotgun and paired-end sequencing runs yielded a total of 2,362,784 and 87,136 reads, respectively. Using the Newbler version 2.7 software program, all these reads were assembled into 26 large contigs (length >300 bp) with an *N*_50_ contig size of 394,001 bp, an average contig size of 118,080 bp, and a single largest contig of 637,827 bp. The draft genome contains 14 gaps between the contigs. Primers were designed and PCRs performed to amplify the gap regions. The gaps were closed by Sanger sequencing (Life Technologies) and subsequently assembled using the SeqMan software (DNAStar).

*P. suwonensis* strain J1 has a single chromosome of 3,891,720 bp, with a G+C content of 70.24%. The genome was submitted to the NCBI Prokaryotic Genome Automatic Annotation Pipeline (PGAAP) for annotation (4). The genome contains 3,065 coding sequences (CDSs). In addition, 127 pseudogenes and 12 frameshifted genes were annotated, and 50 functional RNAs were identified, including 46 tRNAs, 3 rRNAs (5S, 16S, and 23S), and 1 noncoding RNA (ncRNA).

All predicted genes were compared to the Clusters of Orthologous Groups (COGs) database, using BLASTp with *E* values of 1 × 10^-5^ and filtering at 20% match identity and 90% alignment length (5, 6). The results show that approximately 63.07% of the genes were assigned to specific COGs. Metabolic pathways were analyzed by the bidirectional best-hit (BBH) method on the KEGG Automatic Annotation Server (KAAS) (7), and 69.1% were involved in 150 predicted metabolic pathways.

### Nucleotide sequence accession number.

The complete genome sequence of *P. suwonensis* strain J1 has been deposited in GenBank under the accession no. CP011144. The version described in this study is the first version.
